# Supplementary figures and images for: Digger wasps Microbembex monodonta SAY (Hymenoptera, Crabronidae) rely exclusively on visual cues when pinpointing their nest entrances
Source: PLoS One. 2023 Mar 29;18(3):e0282144. doi: 10.1371/journal.pone.0282144 (PMC10058119; doi:10.1371/journal.pone.0282144)

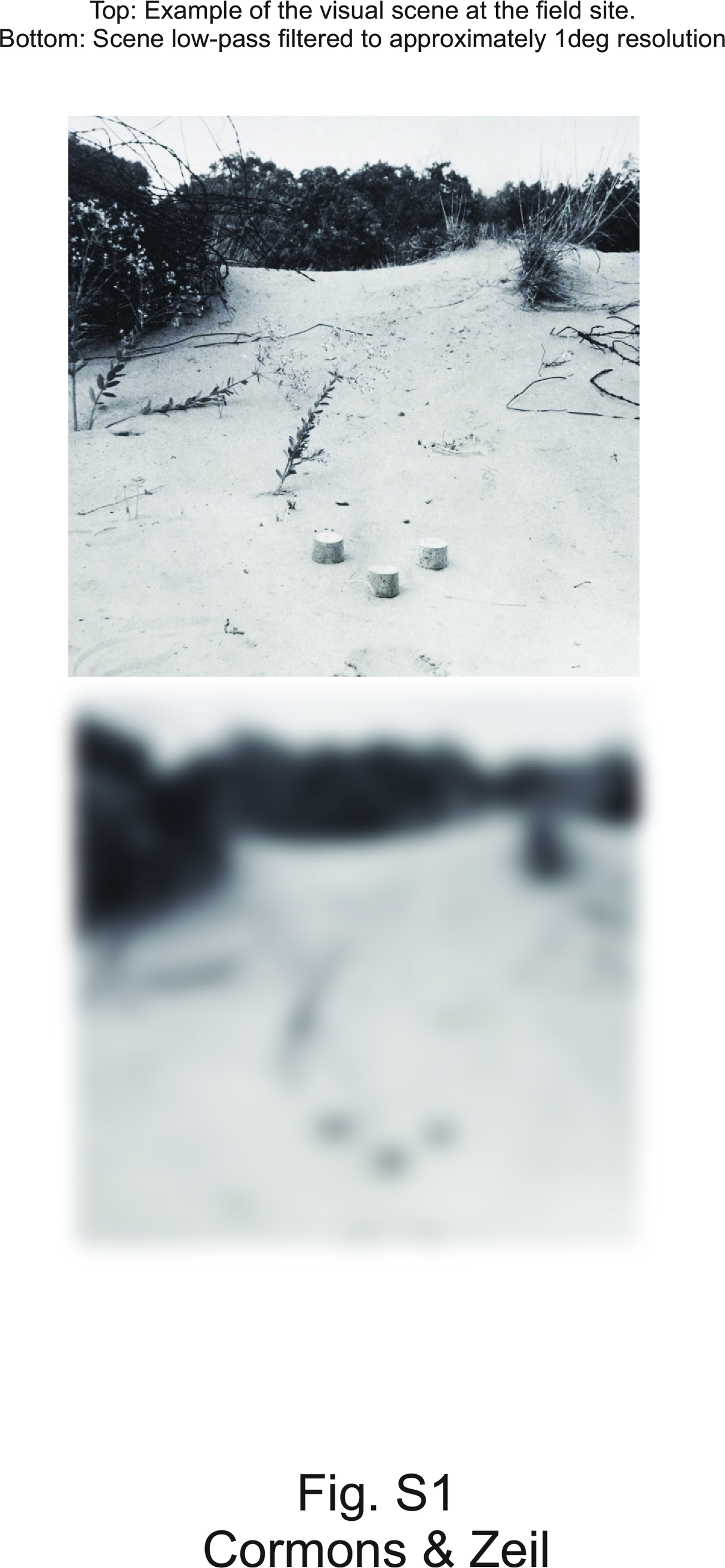

Supplement: S1 Fig — (TIF) [file pone.0282144.s001.tif]

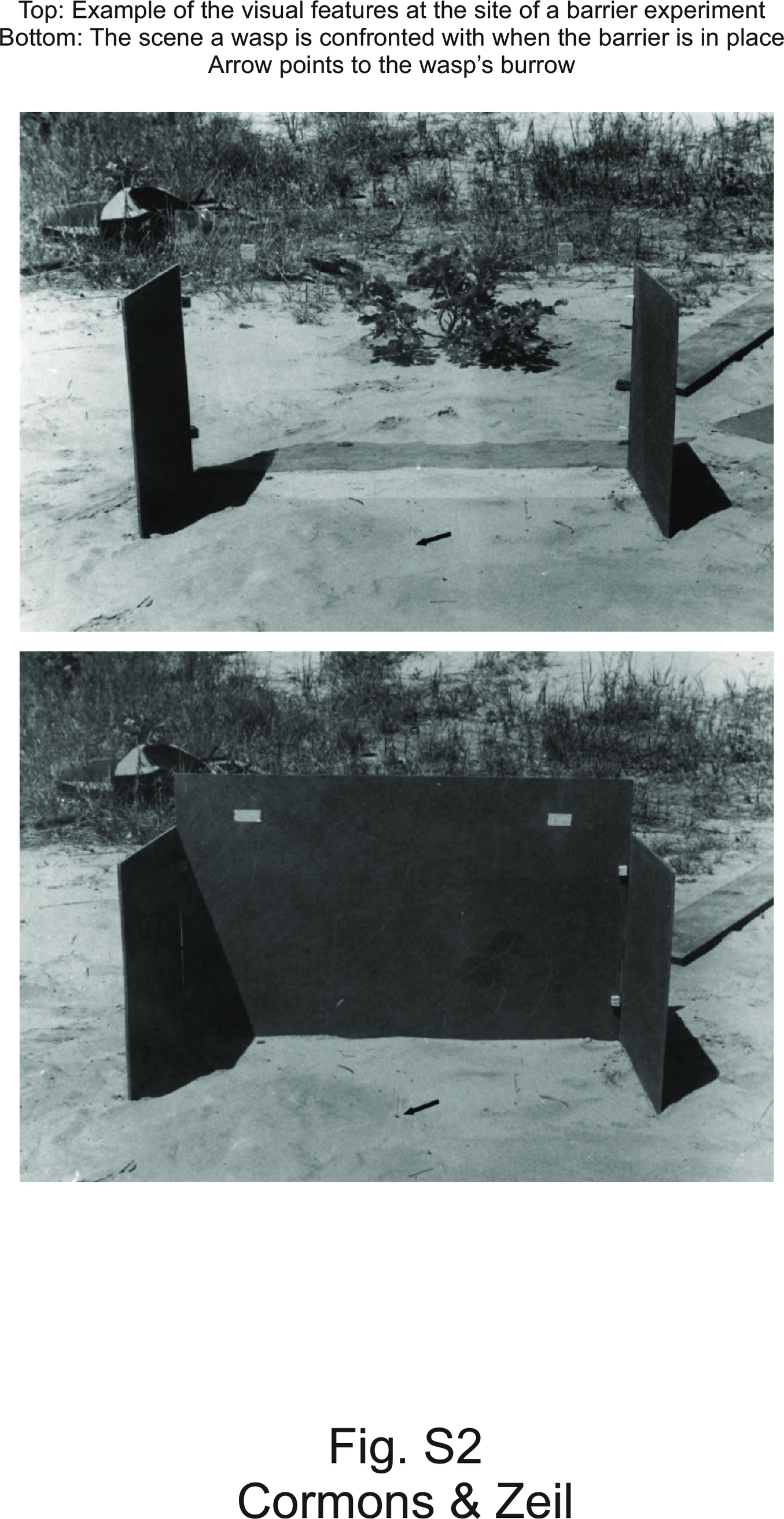

Supplement: S2 Fig — (TIF) [file pone.0282144.s002.tif]
