# Supplementary material for: Digger wasps Microbembex monodonta SAY (Hymenoptera, Crabronidae) rely exclusively on visual cues when pinpointing their nest entrances
Source: PLoS One. 2023 Mar 29;18(3):e0282144. doi: 10.1371/journal.pone.0282144 (PMC10058119; doi:10.1371/journal.pone.0282144)
Supplement: Attachment — Submitted filename: 2022-09-11 CormonsZeil Response to referee comments.docx [file pone.0282144.s005.docx]

*Reviewer #1: The authors provide a series of interesting experiments to study the scale and type of cues used for nest recognition in the digger wasp Microbembix monodonta. I applaud the authors for their extensive revisions, which made the manuscript a focused and clear piece of writing.

I find the experiments and results inspirational (for instance to engage students with in a field course), and especially the results regarding the absolute need for a panoramic cue intrigues me.

I have several minor comments to pinpoint things that are unclear (or not directly clear) to me which need some clarification, or suggestions that would ease the reading for me (or possible typos). The part that deserves the most clarifications, although improved already a lot since the first draft, is the influence of non-visual cues (see comments below), especially what the a priori expectations are of the different materials in the conditions.

My major concern is the representation of the data and statistics (see below), which the authors should consider carefully and find a solution for, fitting the overall aim of their manuscript (I give some suggestions below).

I think the authors will swiftly be able to incorporate my comments and suggestions, as they already made big improvements and incorporated the previous review comments appropriately.*

**Response:** We thank Femke Batsleer for her meticulous referee work and her constructive comments. We have taken all her comments and suggestions on board as outlined in detail below (**line number references are to the TrackChange version of the revision**), but would very much like to keep the descriptive statistics in Fig. 5H for the reasons listed below.

*Major comment:
Although the authors partially met the previous review comments regarding statistics and data transparency, my concerns have not completely disappeared.*

*First, the data presented, with very low sample sizes and repeated measurements on individuals, is in my opinion only suitable for a qualitative description. The authors also mainly write in such an approach, but to be completely transparent about it, the abstract and introduction should also mention the study is qualitative and/or descriptive.

The statistics provided for testing of non-visual cues does not seem appropriate and does not provide real significances (notched boxplots are compared visually, which can only point towards a possible significant difference, especially when comparing 8 groups). At least a one-way ANOVA should be applied, with post-hoc tests to check which groups are different. Preferably, adding a random effect for the individuals tested (as the measurements are not independent). However, there is probably not enough power for the latter. Apart from doing a proper ANOVA, another solution would be to omit any attempt of statistics (also omit the boxplots or only boxplots without grey area or notches), and be purely descriptive in the results and discussion (the discussion regarding the flexible cloth and sand surface could then be retained, but the part about spread in y-direction L453-455 can be omitted). In my opinion, this part is also of minor importance compared to the next experiment (air-tight cover), which is the most conclusive experiment regarding other possible cues.*

**Response:** We would very much like to keep Fig. 5H for the following reasons: (1) The main and surprising result of this experiment is that the wasps can pinpoint the location of the hidden burrow to within a 12cm radius, regardless of any manipulation of local cues. (2) This fact is visually obvious from the size and location of search distributions as indicated by the ellipses. (3) The boxplots show these distributions in a quantitative way as distance of means to the true burrow location (centre location of ellipses) and as the extent of spread (size of ellipses). (4) Notched box plots are an accepted and honest piece of descriptive statistics that allows for a quick visual assessment of the shape of distributions and their differences. (5) The main insight is that these distributions are essentially very similar and a testimony to the astounding accuracy of the wasps. It is practically impossible and not very informative to find functional interpretations for the slight differences we can observe (e.g. medians of y-direction distributions differ by less than 1cm! Medians of spread distributions differ by less than 6cm!). (6) The boxplots also allow us to make data available (Supplementary S3) that can be used for further statistical analyses, if anybody feels that there is more insight to be gained.

*Minor comments:

L23-25: Make this sentence singular, as the species name is in singular (it also broadens the results). E.g. ‘Here we show that a ground-nesting wasp Microbembex monodonta locates its hidden burrow entrances with the help of… only if the view of the wider panorama is not blocked.*

**Done**

*L35 (and many others): delete ‘e.g.’ when citing literature. You always cite examples and can never be complete in a strict sense. Thus, also omit on L39, 44, 48, 340-342, 459*

**Done**

*L37 (and many more): I would try to avoid the term ‘utilize’ and replace it by ‘use’. Utilize holds more the notion of making effective use of objects or a process leading to a product, often beyond its original intended use. The term ‘use’ is more neutral when describing an organism doing something (less anthropocentric).*

**Done**

*L41: can you sum up a few of the groups here (probably similar to L340-342 then). For instance: ‘Vision in homing and pinpointing nests have been reported for other Hymenoptera and insect groups, such as …’*

**Response:** We removed reference to other insect groups at this point.

*L44: not clear what ‘Marchand in…’ means, now it looks like referring to Marchand in all the manuscripts listed there, if it is a chapter in only the first, I would adjust the reference itself into a reference to the book chapter (instead of the complete book).* [*https://guides.library.uq.edu.au/referencing/apa6/book-chapter*](https://guides.library.uq.edu.au/referencing/apa6/book-chapter)

**Response:** Added page reference.

*L47: reformulate ‘shifted introduced cues’: when first reading the sentence I didn’t understand what was meant, as the introducing of the cue happens before the shifting. Suggestion: ‘wasps followed introduced cues when these were shifted,…’*

**Done**

*L58: ‘large screens’: make clear that this is for obstruction of the landscape (and not in the sense of digital screens).*

**Done**

*L65: CO2, 2 subscript, not superscript.*

**Done**

*L68: do you have a reference for this auditory cue of moving larvae as a cue?*

**Response:** No, but larvae are likely to move and eat and this MUST create vibrations and airborne sound. To our knowledge, this has not been investigated in ground-nesting wasps.

*L71: species is singular, so formulate sentence as singular (‘…is a solitary digger wasp that nests in…)*

**Done**

*L74: ‘visually impossible to locate… probably to the wasp itself’: I would omit ‘and probably to the wasp herself’, this is quite a big jump/assumption and very anthropocentric (‘vision’ is anyway something completely different for an insect than what we can sense). The whole manuscript is about nest recognition and how it is indirectly not invisible to an individual wasp (through cues).*

**Done.**

*L75: repeat reference 63 here, to state that closing burrows is an anti-parasite behavior.*

**Done.**

*L79-L83: an introduction should end in research questions and/or hypotheses, not a conclusion of the results. Reformulate to research questions.*

**Response:** The section now reads (line 81ff): “Here, we report on a series of experiments, asking whether *Microbembex monodonta* wasps locate their hidden burrow entrances relative to small local landmarks and whether the view to the wider panorama is needed for pinpointing the burrow. By studying the wasps’ search when their nest entrances were covered in a variety of ways we also tested whether local visual, olfactory and auditory/vibrational cues help wasps to locate their hidden nests.”

*L90: it’s actually quite important that longitude is West (so -89.579446). I suggest to write it as one of the following: (46.172763N, 89.579446W), (46.172763, -89.579446)*

**Done.**

*L104: ‘trained’: what does this actually refer to? If it is was is described in the previous graph, indicate there what the training phase is. Suggestion (if I am correct in interpreting what the training is): L100 rewrite as ‘Each wasp was allowed to return undisturbed, enter, and leave. This is the training phase of the wasp to the objects.’*

**Response:** Done. We added (line 103ff); “This is the phase in which the wasp is trained to recognize the objects.”

*L114: Refer to fig 2 here (if I’m right).*

**Response:** No. Fig. S2 is appropriate.

*L124: make sure to split the sentences with commas if a part is some form of a dependent clause. It’s of course partially a personal taste, but I like it when the writer guides me in where to ‘pause’ when reading (you have to assume as a writer that readers are lazy). The pauses are often obvious to a writer, but this is not always so for a reader. I tried to indicate it in the comments where I think extra comma’s or hyphens (or rather em dashes) would have eased my reading: take these as suggestions. For instance, here, I would a comma between ‘foraging’ and ‘a triangular’. Please also keep this in mind when re-reading your manuscript to add these where confusion with clauses might be possible.*

**Done**

*L127: I wonder if the wasp was not confused already then by the experiment, because you removed already the local cues she probably used to replace them by your own?*

**Response:** The sentence now reads (line 131): “The wasp was allowed to get used to this disturbance and to return, enter, exit, and cover her burrow.”

L132: add something to explain this results in 6cm to the left of the burrow.

**Response:** We are not sure what the referee means here.

*Influence of non-visual cues, experiment 1: this part already improved a lot in clarity compared to the previous version. I have some additional comments/suggestions to add to the clarity:*

*- Already refer in the beginning of this section to table 1
- State in the beginning what the aim is (explore different types of possible cues?)
- State what your expectations are a priori for each of the different materials. This is somewhat deducible from table 1, but deserves more explicit, elaborate explanation in the text (maybe somewhere around L140?). This would also make it more clear, step by step, to the reader how the experiment tests the different types of cues.*

**Response:** The section now reads (line 141ff): “Experiment 1: This experiment was designed to mask or alter local homing cues within the burrow vicinity - visual, olfactory, tactile, and possibly auditory/vibratory (Table 1). The distribution of repeated digging attempts of returning wasps was recorded with the expectation that the absence of crucial cues would lead to no or more widely distributed digging attempts. Seven different covers were placed over the burrows of 12 wasps, each designed to mask or alter at least one potential homing cue that might help the wasps to precisely locate their burrow entrances. Each cover is referred to as condition and the name of each condition begins with the part closest to the land surface, followed in upward placement order of the subsequent parts. The conditions are shown in Fig. 1.”

*- L150 and further: as in the explanation for ‘condition (A)’, state which cues are excluded/included for each condition. It is summarized in table 1, but some written out explanation in the text and captions should complement this.*

*- L159 and further: as previous, state the hypothesis or what is excluded/included and elaborate. It is summarized in table 1, but in the text you can then give more explanation to for instance, the question marks in the table.*

**Response:** We now add this information for each condition.

*- L163: plexiglass should be in italics*

**Done**

*- L168: what is exactly the purpose of this hole?*

**Response:** The hole allowed the wasps access to potential olfactory and auditory/vibrational cues. We point this out now in line 186ff: “This would enable any olfactory and auditory/vibrational cues to emanate, unlike the sealed window in condition (C)). This condition thus masked other visual and sand relief cues, but left olfactory and auditory/vibrational cues unmodified.”

*- L160, 161: 256 or 49 3 cm squares are hard to imagine, I would prefer 16 by 16 (or 16×16) squares with sides of 3 cm. Same for 21 cm² on L161; L164: give exact dimensions of fiberboard frame; give diameter of what is cut from the centre (hole or square?); L174: 256 3cm squares: give the dimensions as well.*

**Done**

*- L160: 11.0 is also in inches? And could you rescale those to cmxcm?*

**Done**

*- Table1: 1) the uncertain states deserve more explanation, as in a previous comment, explain more in the text. 2) for E: cloth alone hugging surface: why is olfactory ruled out for this one? Deduced from the other conditions, I thought this was the one making the olfactory possible. And why would the cloth be masking the auditory cue?*

**Response:** We realize that we probably had been too cautious about the effects of different covers on potential cues and have simplified Table 1. We now clarify the expected effects on potential cues by the different conditions in the method section line 168ff, added caveats where we cannot be absolutely certain about effects and have changed Table 1 accordingly. We also clarified expected effects in Fig. 5. Basically, Conditions A & B have a plexiglass cover which would seriously interfere with auditory/vibrational cues; D would allow both olfactory and auditory/vibrational cues to emanate from the hidden nest entrance and in conditions C & D the area was covered with paper, there would thus be no visual cue from the sand relief.

*L176: omit ‘to me’*

**Done**

*L182: omit ‘for me’*

**Done**

*L203: repeat the objective here (as it was not explicitly stated for experiment 1). But I would not say it is similar to it, but rather an alternative to see if only visual cues are enough to pinpoint the nest.*

**Response:** We now state the objectives at the beginning of the Experiment 1 section.

L206: give exact dimensions instead of squared centimeters.

**Done**

*General for results: state at the beginning of each part shortly what was tested again (readers are lazy), either by explaining it in the first sentence or adding a shot title (rather than “experiment 1”). For instance, on L229: ‘Figure 3B show the responses of one of the wasps to visual barrier that blocks distant visual cues.’*

**Response:** We now have added explanatory sentences to each of the experiments in the results section.

*L233: ‘some’ is quite vague, can you quantify? [now line254]*

**Response:** Sentence deleted.

*L253: explain a bit more in the material and methods (with LXX) that you moved the barriers. Here, in the results section, it comes out of nowhere.*

**Response:** Method section now reads (line 117) “The barriers were at least 60 cm high, but differed in configuration for each of the wasps and could be moved sideways to cover or uncover different parts of the panorama.”

*L285: omit ‘of the’ (ellipses)*

**Response:** We reworded (line 314ff): “Greater homing accuracy is shown by smaller ellipses centred on the burrow entrance location.”

*L346-348: I don’t really understand the azimuth/translation explanation. What does this mean for the orientation of the wasp, does it first set the angle correct and then approaches the goal? Can you elaborate on this?*

**Response:** We now clarify (line 380ff): “ that minimized first the azimuth mismatch between remembered and currently seen objects by changes in orientation and subsequently the apparent size mismatch by moving toward objects that appear smaller than remembered and away from objects that appear larger than remembered.”

*L349-352: is this then the generalized model (as mentioned in the introduction; ‘global image matching’)? Does this one then expand on the snapshot model (as mentioned in the introduction), or is it a contrasting/competing model? Some extra explanation what the difference between both is could be helpful here. Then also end this paragraph with stating that your results support the latter model more. Rewriting the part in the introduction and discussion regarding snapshot and global image matching might be needed to synchronize both parts.*

**Response:** The difference is captured in the two sentences at the end of this paragraph: the original snapshot model assumed that the visual scene can be segmented into discrete individual objects. This is difficult in complex natural scenes, but may not be needed, because global image differences provide the same information. These are not mutually exclusive models: they both operate on visual information that is in principle available to the insects.

*L361: comma between ‘rings’ and ‘found’*

**Done**

*L365: em dashes, or commas, after Tinbergen and between ‘triangulum’ and ‘found’.*

**Done**

*L369: what is ‘motion parallax’? When introducing a new term, elaborate on what it means and why it is relevant for your study.*

**Response:** We avoid using the term motion parallax, but rather explain what it means directly (line 403ff)“: as a homing insect moves closer to the goal, the images of close objects move faster across its visual field than those of more distant objects”

*L393: ‘to home more easily/rapidly’?*

**Response:** changed to “more efficiently”

*L414: a dot to much at the end of the sentence.*

**Done**

*L452: ‘in front’ instead of forward (I was misguided by the ‘forward’ term as I first interpreted this as an actual overshoot).*

**Done**

*L455: I doubt you can talk here about ‘significantly’ different from A, as the ‘statistical’ comparison is only with A (see major comments); if the spread of B is indeed higher than others, my first idea would be to think about the multitude of material layers added to this condition (or the frame, the spread seems high for the conditions with the frame, eyeballing the ellipses in Fig. 5).*

**Response:** Changed to “possible”.

*L467, 469, 475: replace utilize by use*

**Done**

*L469: put ‘:’ after ‘as such’, because the next sentence explains the ‘no functional distinction’.*

**Done**

*I would also nuance this statement more, because you did not test homing to the broad burrow vicinity, but only that in the burrow vicinity, a panorama is crucial for locating the nest entrance (which is a very interesting thing in itself). So I would not make a conclusion stating that homing to the nest aggregate is functionally similar to local nest recognition, as the former has not been tested. It probably uses landscape features as well on a larger scale, but this has not been tested. I suggest to omit this statement, and as a replacement stress the importance of the surrounding landscape.*

**Response:** We modified this sentence (line 504ff): “There may thus be no functional distinction between homing to the broad burrow vicinity (although this remains to be investigated) and recognition of the burrow location as such:…”
