# Supplementary material for: Digger wasps Microbembex monodonta SAY (Hymenoptera, Crabronidae) rely exclusively on visual cues when pinpointing their nest entrances
Source: PLoS One. 2023 Mar 29;18(3):e0282144. doi: 10.1371/journal.pone.0282144 (PMC10058119; doi:10.1371/journal.pone.0282144)
Supplement: Attachment — Submitted filename: 2023-01-27-CormonsZeil Response to referee comments.docx [file pone.0282144.s006.docx]

I am grateful to both Volker and Femke for their diligent reading of, and their constructive comments on the manuscript. I hope to have comprehensively addressed them as indicated below, with the biggest change being a statistical analysis of the results presented in Fig. 5. **Line numbers refer to the TrackChange version of the manuscript**.

Sadly, my co-author Matt Cormons, who did the experiments presented in this manuscript has passed away in early December last year.

**Comments by Volker Nehring, Academic Editor:**

*I have read the manuscript myself and had the same problems as the reviewer – I am not convinced by the notched boxplots for a number of reasons, some of which are trivial, others not.
1) For starters, the way the figure is set up will lead to the botchplots being very small on a page – it will be difficult to properly assess overlap
2) as the reviewer pointed out, dependency of the data points in the different groups (repeated measures) are nothing notched boxplots take into account. I would actually think that taking this into account will make the differences even more clear
3) notched boxplots are not made for multiple comparisons. In theory you compare each of the boxes with each of the others, which would require some serious adjustment of p-values. We just can’t interpret notches like that. I do think that the way you set this all up is the best possible solution for these kinds of problems – you actually compare all treatments to a single (no-cue) control. However, in combination with the low sample sizes I do not think the presentation of the results purely based on the notched boxplots is appropriate and I would urge you to revise it.*

*There are multiple possible ways to improve the presentation of the data, for example:
- a repeated measures anova or a Friedman test would at least give us information on whether there are any differences between the treatments. Based on this I would bemuch more inclined to trust the box plots
-I would actually favour plotting individual data points with such a sample size
the summary output of a linear model in R would actually test each treatment against a single control – which would be fitting if that control was the treatment that did not offer any cues.*

**Response:** It is important to clarify that in the experiments shown in Fig. 5, the information on wasp IDs and the location of individual digging attempts has been lost, but that otherwise the data set is balanced, because each wasp contributes the same number of data (mean and spread of 15 digging attempts) to each condition and each of 12 wasps was confronted with each condition. This has now been clarified in line 210ff.

Linear model results are now tested with ANOVA and in addition with a permutation test using the F-statistics of the linear model as the permutation statistics, both showing that there are no significant differences in how wasps respond to the different conditions. A t-test was employed to test differences between the x- and y-directions of means and spread across all conditions, which are significant.

Statistics are now described in line 214ff and results are presented in line 354ff.

In addition, individual data points are now shown for each conditions, together with their median**.** See Fig. 5H and legend line 378ff.

*Additional Editor Comments:

I had some further comments, please see below. Importantly, I often had problems to understand what the actual data points were

line 43 “to precisely locate their burrows. The shifting of the latter causes homing wasps”. That sounds like you mean digging within the burrows*

**Response:** The sentence now reads (line 43ff): *‘The shifting of such local objects causes homing wasps to dig for their burrows relative to the shift…***’**

*67topography or even the sound of moving larvae emanating from the nest may help ground-nesting insects to pinpoint the exact location of the entrance. Any reference for the larval sounds? If not, please make it more obvious that this is your own speculation*

**Response:** Now referring to (line 67): Müller A, Obrist MK (2021) Simultaneous percussion by the larvae of a stem-nesting solitary bee – a collaborative defence strategy against parasitoid wasps? Journal of Hymenoptera Research 81: 143–164. https://doi.org/10.3897/jhr.81.61067

*137 5) The influence of non-This should be 4), shouldn’t it?*
**Response:** Corrected.

*Fig 3a: I don’t understand what its shown. According to the text these figures are the attempts of a “typical wasp” – but only five wasps were tested and 6 wasps are shown? I also don’t understand the explanations for f, g and h. The figure caption implies that the wasps always dug where the cork configuration would indicate – but it seems b, c , d and h did not dig in the centre of the triangle? Was the triangle not centered around the burrow? Where were the burrows previously? It can’t be the black dot because in c the dot does not line up with the x either. There is really too little information for me to understand what is shown and what the data points are.*

**Response:** Figure 3 has now been simplified and a better description is offered both in the legend and in the text:

**The figure legend now clarifies (line 269ff): ‘***Fig. 3 - The influence of local and distant cues. (A) A typical example of how one of 5 wasps responded to shifts of local landmarks. Three cork stoppers (gray circles) were introduced around the burrow entrance (black dots) of a wasp. (a) training trial for the wasp to learn introduced cork-configuration cue surrounding the burrow prior to first shift. (b) – (h) consecutive configuration-shifts and digging attempts (⊗) of this particular wasp, which eventually does find her burrow; each initial digging following a subsequent shift was where the wasp was last successful, relative to the configuration. At (g) the wasp was not permitted to find her burrow before the next shift, resulting in her digging relative to configuration in (h) at a location where she had found the burrow in (f). (B) A wasp responding to the presence of an opaque barrier behind the burrow, with digging attempts at either end of the barrier with unimpeded view of the wider surrounding. (C) Same wasp digging at the ends of the barrier when the latter is centered on the burrow, then digging at either end, when the barrier is shifted to the left and right of the burrow.’*

**The text now refers to the simplified figure (line 249ff*):*** *‘Local landmarks influence where wasps search for their hidden burrow entrances (Fig. 3A): Five wasps were allowed to become accustomed to finding their burrow in the center of an array of 3 cork stoppers (‘training’) and on subsequent returns were confronted with a shifted array of these landmarks. In each shift-trial wasps eventually located their burrow. All responded similarly to the one shown in Fig. 3A. Before attempting to dig for their burrows the wasps reacted by flying around the shifted configuration of local landmarks for two to three minutes, almost always where the configuration led them and their initial digging attempt was always at a location where, relative to the landmark array, they had found their burrow on their previous return (see [56]) for more evidence of this rapid location learning). Eventually the wasps corrected themselves and dug at their burrow entrances, which had not been disturbed; they did so by first hovering over the place they had been misled to dig, then flew directly above the actual burrow entrance, landed, and dug.’*

*Similar problem in Fig 3B -the tests says these are the responses of one of the wasps, but in the Figure it seems like these are responses of different wasps.*

**Response:** Figure 3B has been removed, including related section in the legend and in the method section (line 104ff).

*Lines 267-272 Please be more specific here – how many wasps were tested with which of the types of barriers?*

**Response:** line 298 now clarifies that 6 wasps were tested, but only one made digging attempts as indicated in Fig. 3B and C.

*Fig 4: I don’t find it clear what is shown on the x-axis. what does “responding to” mean? Is this the number of wasps searching where the artificial landmarks indicate, or where the burrow really was?*

**Response: Now clarified in the text (line 305ff):** ‘*To investigate how local and distant cues interact when wasps attempt to locate their hidden burrow entrances, their response to small shifts of local landmarks was tested in the presence of a large opaque barrier. Both digging attempts at the center of a three-cube landmark array and hovering above it were considered as an indication that the wasps used the visual appearance of the landmarks to locate their burrow. The barrier set-up for these experiments is shown in Fig. 4A and the sequence of tests with local visual cues in Fig. 4B. Results for all 10 wasps are shown in Fig. 4C. All 10 wasps learnt to use the cube-configuration as a homing cue after a single exposure (Fig. 4B, a). When tested with the shifted cube-configuration (Fig. 4B, b) 8 wasps first dug at the center of the shifted array before locating their burrow. The two that did not follow landed and dug without hesitation at their actual burrows. One of the eight wasps that located their burrow did not do so immediately; she hovered over the point where the cubes misled her. However, she quickly shifted to her actual burrow location, landed, and successfully dug*.’

**And line 330ff:** ‘*Once the barrier was in place, masking frontal, and some lateral, distant cues all 10 wasps failed to dig at the center of the landmark array, none hovered above it and none did find her burrow (Fig. 4C, c). The wasps flew back and forth in front of the barrier, around, and over it (see Fig. 3B), and even flew among the cubes. They never attempted to dig, never hovered over any particular area, nor otherwise indicated any recognition of the cube-configuration; they appeared to be lost. Yet, all 10 wasps had been able to enter the vicinities of their burrows. When the barrier was removed nine of the 10 wasps pinpointed the nest in the center of the landmark array without difficulty; the tenth wasp did not return*.’

**And in the figure legend (line 325ff):** ‘***(C)*** *The number of wasps responding to the shift of the landmark configuration by digging attempts or by hovering at the center of the landmark array in condition (b), (c), and (d) (see (B) above). None of the wasps followed the configuration in (c).’*

*I would also suggest to add statistics to Figure 4C (e.g. a chi-squared test).*

**Response:** I am advised that one cannot do a chi-squared test with zero responses. I do think, however, that the results are very clear without statistics.

*To be honest, after seeing the data in Fig. 4 I wonder if Fig 3 is necessary at all.*

**Response:** The barrier/local landmark experiment shown in Fig. 4 demonstrates that local landmarks can only be used by the insects as a visual aid to burrow location, if they are seen in the context of the full landmark panorama. This is additional information, compared to what has been shown in Fig. 3, namely (a) that local landmarks are attended to, (b) that that screens affect the wasps’ ability to locate the burrow and (c) that they tend to dig at (inappropriate) locations where at least the distant landmark panorama matches their burrow location memory.

*304seven conditions described in the method section and in each of the panels in Fig. 5. Each condition graph shows the areas for 99% of all attempted diggings as grey ellipses and the means of the search. How many digging attempts per wasp?*

**Response:** Each wasp was allowed 15 digging attempts for each condition. This is now clarified in lines 205, 212, line 342 & and in legend l368

*Fig 5 what’s the definition of an outlier here?*

**Response:** Bar graphs are now only shown for all data across all conditions, while the rest of the panels show individual data for each condition. An outlier is a value that is more than 1.5 times the interquartile range away from the bottom or top of the box. Now clarified in legend line 376ff.

*Lines 361-428: A lot of this text feels like a repition or expancsion of what was already said in the introduction. I would find it more appropriate in that section*

**Response:** I’d like to keep this section because it allowed us to discuss the details of present results (such as the influence of local and distant cues) in the context of previous work. This would be difficult to do in the introduction without stressing the patience of readers.

*Dataset S3: Since these are repeated measures it wouold be good if you could add the IDs of the individuals to each line of the data set. In general I it was unclear how many digging attempts were observed per wasp. Perhaps it would be best to also include the coordinates of each digging attempt.*

**Response:** The ID of wasps and the coordinates of their individual digging attempt have unfortunately been lost. This is now clarified in method section line 210ff.

**Comments by Femke Batsleer:**

Reviewer #1: The authors provide a series of interesting experiments to study the scale and type of cues used for nest recognition in the digger wasp Microbembex monodonta. I really look forward to using this manuscript for education and as a reference in my own work regarding nest recognition behavior.

However, there is in my opinion still one fundamental issue with the framing of the statistics used. Notch boxplots are indeed an accepted piece of descriptive statistics, but that does not mean it is applicable to any kind of experimental design, as in this case one with repeated measurements. I give suggestions below how to frame the statistics used more descriptive and qualitative. I also added some last (very) minor comments. This work is almost ready to be published, but the fundamental statistical concept I point to is too crucial to be ignored (if you don’t use the appropriate statistics for the experimental design at hand, your significance values are unreliable).

**Response:** We now present statistics (line 214ff), but it is important to note that information on wasp ID and individual digging attempts have been lost (line 210ff). Please see my responses to similar concerns by Volker Nehring above.

*Major comment on the use of notch boxplots
Firstly, yes they are indeed an accepted piece of descriptive statistics, but it does not mean it is very well suited to every experimental design. As explained in my previous review, there were repeated measurements involved, but the sample size does not allow to properly take this into account. You can indeed still use such visualizations descriptively, but cannot conclude anything regarding significance as the statistics are strictly speaking not applicable for the experimental design. I would thus suggest to omit any reference to ‘significant differences’ and use the notch boxplots as purely qualitative/descriptive. At the following lines such ‘significance’ is mentioned: L308-309, L319-321, L466, L476. Please omit these and be more descriptive about it (such as ‘indications for’, ‘seems to be’, ‘there is a visual difference in distribution’, etc…). Then as we cannot talk about ‘significant’ difference with this experimental design/statistics, omit the part in the discussion on the second difference (Condition A, compared with Condition B, D) L477-479, as you mentioned in your response that the difference is only 1 cm. Of course, in the results you can still add that there seems to be a small, probably negligible, difference for that case. But it’s not worth mentioning in the discussion when there is nothing to discuss about.*

**Response:** We now do present a statistical analysis (linear models, permutation test and t-test) that shows that the null-hypothesis, namely that the responses of wasps to the different conditions all come from the same distribution cannot be rejected (line 353ff). Which makes any comparison of responses to individual conditions superfluous (line 522ff).

*Secondly, what you described in your responses, that the main insight is that these distributions are essentially very similar, was an eye-opener for me that this should be the main focus of the results and not the few small, probably negligible, differences. It is a very important interpretation that should be integrated in the text itself, I would suggest in the results (in paragraph L314-322) and in the discussion on L466.*
*Thus, in conclusion, shift the focus in both results and discussion to the fact that these distributions are essentially very similar, but there does seem to be a difference with condition E and F which has a reasonable explanation, which you discuss. Omit any notion of significance in both results as discussion as you can only apply descriptive statistics and discuss the distributions qualitatively.*

**Response:** See response above and text line 522ff.

*Minor comments:
L22: Hymenoptera with capital*

**Response:** Corrected.

*L67, L448-453: as you mentioned in your previous response, this is a hypothesis of yourself and very suggestive. State it as such if you don’t have any reference to back-up such a statement. But maybe there is some literature out there that points to sounds of larvae of insects being a cue to conspecifics (beetles, social wasps?)?*

**Response:** Reference added ([53] Müller A, Obrist MK (2021) Simultaneous percussion by the larvae of a stem-nesting solitary bee – a collaborative defence strategy against parasitoid wasps? Journal of Hymenoptera Research 81: 143–164. doi.org/10.3897/jhr.81.61067

*L71: ‘…is a solitary…’*

**Response:** Corrected.

*L118: ‘check’ instead of ‘ask’ (conducting an experiment is the step after asking a certain research question).*

**Response:** Corrected.

*L164, 170: 21.6cmx27.9cm*

**Response:** Corrected.

*L172: insert enter before condition C*

**Response:** Corrected.

*L216: omit one dot (two dots present)*

**Response:** Corrected.

*L218: ‘…similar to the…’*

**Response:** Corrected.

*L221: 5.7cmx5.7cm*

**Response:** Corrected.

*L268: space between ‘C,D’*

**Response:** Corrected.

*L301: excluding instead of exclude*

**Response:** Corrected.

*L305-313: this is explanation about a method you used, so move this to the results section. Then the results are immediately about what you see in the figure. The explanation in the caption of the figure itself can stay.*

**Response:** You probably meant moving this to the Method section. I’d prefer to leave it here, so that readers do not need to go back to the method section.

*L364: add closing bracket ‘)’*

**Response:** Corrected.

*L383, 410, 501: the ‘van’ Iersel and ‘van den’ Assem are actually part of the surname (they are Dutch names), so it should actually read (also in the reference): ‘van Iersel and van den Assem [10]….’*

**Response:** Corrected.

*L413: ‘A’ should not be a capital; 30cmx50cm*

**Response:** Corrected.

*L503: please add references to this final statement (what are the recent experimental and modelling evidence you talk about?).*

**Response:** Review added [65] Zeil J. Visual navigation: properties, acquisition and use of views. Journal of Comparative Physiology A 2022. doi.org/10.1007/s00359-022-01599-2
