# Supplementary material for: Digger wasps Microbembex monodonta SAY (Hymenoptera, Crabronidae) rely exclusively on visual cues when pinpointing their nest entrances
Source: PLoS One. 2023 Mar 29;18(3):e0282144. doi: 10.1371/journal.pone.0282144 (PMC10058119; doi:10.1371/journal.pone.0282144)
Supplement: Attachment — Submitted filename: 2022-04-13_Response to reviewer comments.docx [file pone.0282144.s004.docx]

We thank reviewers for their constructive and positive comments. In this revision, we took most of their suggestions on board, which we detail below. In particular:

(1) We changed the title (ref. 1).
(2) We shortened the abstract to concentrate on outcomes and interpretation of results (ref. 1).
(3) We completely reworked the introduction and the discussion to put our results into the perspective of recent work on insect visual navigation, homing and nest entrance localization (ref. 1).
(4) We added statistics to the outcomes of the non-visual cue experiments (ref. 2)

Detailed responses to reviewer comments:

Reviewer #1: The authors present a series of inventive experiments to research the cues (local, distant, possible non-visual cues) related to nest recognition in the digger wasp Microbembix monodonta. I appreciate that the authors wrote this manuscript from data collected almost 50 years ago. I admire the perseverance and courage needed to do this and it deserves a place in the scientific literature.
However, the manuscript needs profound rewriting/restructuring, rethinking of term use and reframing of the experiments in light of the current scientific literature, as the research is somewhat outdated. Nevertheless, if the manuscript is rewritten, this would be a nice natural history publication. I see room for turning the disadvantage of reporting old experiments into an advantage by highlighting the need for natural history research to set the base for current behavioral and ecological research. This type of research is becoming rare but remains valuable. I really want to motivate the authors to rewrite their manuscript because renewed interest for this kind of work is needed, but in an accessible format for current researchers.

**Response:** Thank you and we appreciate your thoughts here. Rather than highlighting ‘natural history’ (which we do think is very important and much neglected) we rather chose to frame the experiments in the context of recent research in insect visual navigation, so more in a neuroethological context.

The data is quite hidden in text and figures with no clear overview of the data itself. The data should be made available in a more direct format (e.g. tables).

**Response:** We now make the data for the non-visual cue experiments available as an Excel spread sheet.

In depth suggestions and comments are given below.
I am looking forward to give more detailed comments on a revised manuscript.

Major comments:
I find the term use ‘homing behavior’ too broad for how it is used in the manuscript. This probably stems from how this term was used in for instance Tinbergen’s papers, but its meaning has expanded in the meantime. Homing behavior is currently used mainly for large distance orientation back to a nest site (Schöne & Tengö, 1991; Tengö et al., 1996; Goulson & Stout, 2001; also see ornithological research). Nest recognition (see for instance Plowright et al., 1995) can be seen as part of this, but they are not synonyms.

I advise to rewrite the manuscript by being more specific in what is studied in this manuscript, for instance using the term ‘nest recognition’. Homing behavior should then be added to the key words.

**Response:** We use the term ‘pinpointing the nest entrance’ now to be clearer and in line with the terminology used in the more recent insect navigation literature. We have reworked both abstract and the introduction to put our work in the context of recent research in this area (details below).

Title: related to the previous comment, I would use something more specific and a title that says something about the results of the manuscript rather than a title that is a very broad topic in itself. A suggestion: ‘The digger wasp Microbembix monodonta uses visual cues in nest recognition’.

**Response:** Title now reads: DIGGER WASPS *MICROBEMBEX MONODONTA* SAY (HYMENOPTERA, CRABRONIDAE) RELY EXCLUSIVELY ON VISUAL CUES WHEN PINPOINTING THEIR NEST ENTRANCES.

Abstract: I think there are too many details here regarding the methods and experiments. As a reader without any background on the story, I was a bit lost regarding the bigger picture and where this was headed.

Shorten the abstract and mention what type of experiments were performed, but don’t give details how exactly. Focus on the outcome and interpretation of the results. Currently, methods and results and interpretation are presented mixed. Make a flow more parallel to the paper (intro-methods-results-interpretation/conclusion) in the abstract as well.

Some suggestions: L14-L19, shorten this into one or two sentences with, for instance, following structure: “Returning wasps were monitored where they attempted to dig when either one or a combination of local cues and visual barriers were introduced.” L19-26: shorten this likewise with the potential olfactory, auditory and tactile homing cues as focus. Then in a next sentence, summarize the results and then give your conclusion.

**Response:** Done.

The writing is in a style that is very focused on the author (sentences often start with ‘I…’ and ‘my…’). In general, I do like a more active style in manuscripts, as writing too passively often makes it more boring. However, here, it is often distracting from the main topic or experiments.

I advise to rewrite such sections by putting the stress on the subject of the experiments rather than the author who performed something. (See for instance: L45, L55, L60, most subsections in the methods,… L257,…).

**Response:** We followed the advice throughout with a few exceptions, were an active form was more appropriate. We are now also co-authoring the paper.

There is a lack in discussing this research within a recent scientific framework. How is this research relevant for current behavioral and movement ecology, evolutionary biology,…? Both introduction and discussion could use embedding in a more modern framework. I think a good starting point to stress the value of your work would be in the importance of natural history in current ecological and evolutionary research, see for instance Travis (2020).

**Response:** We now review current concepts and research background on insect visual navigation, including pinpointing goals, such as nests, in the introduction and discuss our results within the current frame work in addition to the extensive older literature.

It should be more clear in the introduction (or methods) what the exact aim is of the visual barriers: is it to obstruct local cues so they should only rely on more distant cues? Or rather that they only see the local ones when they are already close to the nest? This should be explained somewhere in more detail.

**Response:** We now clarify in the introduction (line 48ff): “ Cartwright and Collett [25,42] have shown that this search behaviour can be explained by assuming that insects memorize the view from the nest entrance (as if taking a snapshot) and upon returning to the nest area moving in such a way as to minimize the mismatch between what they currently see and their memorized snapshot. This snapshot model of homing can be generalized to global image matching without identification of individual objects in a panoramic view from the nest (3,5,43). The crucial insight here was that a location in a natural environment is uniquely defined by the view taken from it because global image differences – in the simplest way measured as the root mean squared pixel difference between a memorized goal image and the images seen on approach to the goal – become systematically smaller as the distance to the goal decreases. Pinpointing the nest entrance, thus becomes a gradient descent in global image differences [43,44, see also 45-51] and experiments with large screens have added to other evidence (see examples in [1]) that navigating insects do make use of the full visual panorama and not only of individual landmarks [9].”

The results are quite hidden in the text and figures. It might be useful to give results in a table (how many wasps of the total had what response; even if it is one row for one table). Especially because now I have to look back and forth for the number of wasps for a certain response and total numbers. And such results are in need of some basic statistics: chi-squared tests should give a basic idea if the number of wasps is different for the different response groups. These statistics could be put in the same table.

**Response:** (1) We now clarify in the text and in figure legends that Fig. 3 shows individual examples of outcomes observed in x number of wasps (2) We added numbers to the bar graph in Fig. 4C and (3) now show the results of the non-visual cue experiment in the form of notched boxplots in Fig. 5H together with wasp and sample numbers, which offers an immediate visual assessment of significant differences in the search distributions. This was also requested by refer 2. We also re-drew the side-view insets to improve clarity.

Discussion: a lot of new context or terms are introduced here (e.g. retinotopic matching, the use of local and distances cues). I would suggest to already move some of these things to the introduction, to make the introduction and discussion work in parallel. As the introduction is now very short, some shifting of the body from the discussion to the introduction would balance this more.

**Response:** We introduce current concepts of visual navigation based on image matching in the introduction including open questions regarding the role of local and global cues in pinpointing goals. We then can refer back to these concepts in the discussion of our results.

Structure of discussion: in general, the discussion feels quite chaotic to me and mainly has to do with the writing structure and the style (e.g. starting a new paragraph with ‘also’, ‘with regard to’).

I suggest to try to integrate paragraphs more, start the paragraph with a sentence that already gives the focus of that paragraph rather than the context (which should be described in the body). Such a structure is more readable for a first reader, especially also for someone who is scanning the paper for interesting discussion topics (they will mainly look at the first and last sentence of each paragraph to find what is useful for them). For instance, paragraph 2 of the discussion: start with a sentence regarding retinotopic matching; paragraph 3: disorientation; suggestion: then a third paragraph on local and distant cues,…

**Response:** We now begin the discussion with a summary of results and a brief introduction into the issues to be discussed. We have substantially reworked the discussion to create a more logical flow.

Conclusion: new discussion topics are brought up (around L382, and especially from L385 onward), which should be integrated in a paragraph in the main discussion regarding distant and local cues, rather than starting a new discussion topic in the conclusion.

**Response:** Done.

Writing style in general: it feels a bit outdated, but you can use for instance Plowright et al (1995) as an example regarding general structuring. I don’t see ‘being outdated’ as something negative, it is what it is (I actually also like it in a way when reading manuscripts from the ’60, ’70 etc). But I am convinced the manuscript would be more accessible to, and picked up more by, current researchers if a more ‘modern’ writing style would be adopted. With that, I mainly mean how an abstract is structured, writing style not focused on the first person form, how the hypotheses or research questions are introduced in the introduction, what the first paragraph of an introduction does, etc…

**Response:** We hope that the revised manuscript does now take these issues into account.

Minor comments:
L14: accomplishes

**Response:** Removed.

L45: avoid literally referring to ‘my master’s thesis’, as the manuscript here should be an independent report of the results. I suggest to rewrite this sentence to focus on what is actually done and the research question (with a reference to the thesis).

**Response:** Removed.

L49-50: ‘rendering it invisible’: this feels quite anthropomorphic (in the sense that is invisible to us), but probably with the main function to be invisible to possible predators/parasites (see Poldori et al. (2009) and references therein). As you describe experiments on how it is not (indirectly) invisible to the wasps themselves, I would reformulate this sentence by focusing on the function of closing their nests.

**Response:** Wording changed (line 74): “…then kick sand over the burrow entrance, making it visually impossible to locate, certainly to a human investigator and probably to the wasp herself, as well as to potential predators and parasites.”

L61-63: I would omit this here, and rather give a clear summary of research questions + short what was done in the last paragraph of the introduction.
L63: omit or reformulate (suggestion: ‘Three types of experiments were performed to test the influence of local visual cues, the influence of distant visual cues, the combination of the former two and the influence of non-visual cues.’ Then the rest of the methods follows, but it is already clear for the reviewer what structure or subsections to expect.)

**Response:** The second paragraph of the Method section now reads (Line 93ff): “The following experiments were conducted to test for: 1) the influence of local visual cues, 2) the influence of distant cues, 3) the interaction between local and distant cues, and 4) the influence of non-visual cues.”

L120 and following + fig 1: can you give more meaningful names to the conditions? There are quite a few conditions, and when I try to understand table 1 I constantly have to go back and forth between table and main text. Maybe use the ‘acrylic-paper-cloth-sand’ wording? It’s longer, but more meaningful and would make table 1 more directly interpretable.

**Response:** We now follow the referee’s advice on labelling the conditions**.**

Table 1: this table can use more explanation in the caption (a summary of what is in the text). A table (and figure) should be interpretable on its own without having to read the full text (+ they are the billboards for your paper).

**Response:** We have changed the title, the caption and the condition names of Table 1 as suggested.

Fig.4: C should be given in a table (see previous comment on results in general), then it would be more clear what the total number of wasps were.

**Response:** We now added numbers of wasps above bars and total number in heading.

Fig.5: mention somewhere in the caption what the actual nest is (the yellow dot I presume?).

**Response:** Added to figure legend (line 306ff): “Burrow entrance marked with a white circle at the intersection of x- and y-axes as labeled in G.”

L257-263. Give a summary of all the results and only start the discussion in the next paragraph, as L260-263 is already a very specific discussion point in itself, and should be integrated elsewhere. The first paragraph of the discussion should be a summary of the results.

**Response:** Beginning of the discussion now reads (line 322ff): “The experiments presented here demonstrate that *M. monodonta* wasps very quickly learn to use introduced local visual cues to locate their burrows. When these cues are shifted the wasps’ first digging attempts were always at the location they were last successful relative to the configuration. However, when the more distant visual panorama was blocked by barriers, wasps were unable to locate their burrows, even when the local cues they previously followed were still present. Finally, wasps were able to locate their hidden burrow position to within a few centimeters when all possible non-visual local cues were excluded by different layers of materials covering the nest entrance, including an airtight cover. *M. monotonta*, like many other nesting insects, thus rely exclusively on local visual cues in the context of the wider visual panorama to locate their hidden nest entrances.

These results confirm a large body of literature on the homing abilities of insects, but also raise a number of interesting questions regarding the accuracy of visual homing and the relationship between global visual cues and the fine visual details close to the goal. Lastly, we discuss the question why other, especially olfactory cues do not play a role in nest entrance recognition.”

L337: ‘and those of others with digger wasps’: give references.

**Response:** Removed.

L358: ‘Conditions (F) and (G)’: give more meaningful explanation, as someone reading the discussion might not have read the methods in detail.

**Response:** Section now reads (line 441ff): “The search distributions across the different conditions in which the burrow was covered with different layers of material did not differ significantly except in the following cases: first, wasps searched closer to the burrow location in the direction of approach (y-direction) in Conditions E and F, where covers consisted of flexible cloth and of a flexible cloth over a flexible plastic sheet, respectively. Interestingly, these were the only conditions that hugged the sand surface (0 cm above the surface), indicating that the wasps may have payed attention to subtle differences in elevations of the sand surface. *M. monodonta* approach their burrow vicinity flying very low, about 1 cm above the sand surface, facing forward (toward the cell end of the burrow). Slight hills and valleys in the sand surface might help the wasps in precisely locating their burrow entrances. With the elevated covers in the other conditions, wasps were forced to fly higher than normal and may have not been able to detect elevation differences very well.”

L382: no closing bracket present.

**Response:** Removed.

L391: I like this concluding interpretation/sentence!

**Response:** Thanks, but this had to be moved in the revision to line 380ff.

References
David Goulson and Jane C. Stout (2001) Homing ability of the bumblebee Bombus terrestris (Hymenoptera: Apidae) Apidologie, 32 1 (2001) 105-111. DOI: https://doi.org/10.1051/apido:2001115
Polidori, C., Ouadragou, M., Gadallah, N.S. & Andrietti, F. (2009) Potential role of evasive flights and nest closures in an African sand wasp, Bembix sp. near capensis Lepeletier 1845 (Hymenoptera Crabronidae), against a parasitic satellite fly. Tropical Zoology 22: 1-14
Schöne, H. & Tengö, J. (1991). Homing in the Digger Wasp Bembix rostrata - Release Direction and Weather Conditions. Ethology, 87, 160–164.
Tengö, J., Schöne, H., Kühme, W., Schöne, H. & Kühme, L. (1996). Nesting cycle and homing in the digger wasp Bembix rostrata. Ethol. Ecol. Evol., 8, 207–211.
Joseph Travis (2020) Where Is Natural History in Ecological, Evolutionary, and Behavioral Science? The American Naturalist 196:1, 1-8, DOI: 10.1086/708765
C M S Plowright, Colleen E O'Connell, Lisa J Roberts & Sheri L Reid (1995) The use of proximal and distal cues in nest entrance recognition by bumble bees, Journal of Apicultural Research, 34:2, 57-64, DOI: 10.1080/00218839.1995.11100888

Reviewer #2: The present manuscript by Mathew Cormons describes a series of experiments designed to determine the cues used by the digger wasp Microbembex monodonta to determine the position of its nest. Although the study seems interesting, and the experiments are sound and have been well designed, I am not positive about the news-value of the study or whether it just adds another digger wasp species to the wide literature on homing behaviour in digger wasps. This is mainly due to the fact that the author does not justify his experiments (which could be done in the introduction) to convince the reader that they are really necessary.

**Response:** We now justify the experiments in the introduction with reference to the recent literature and to current concepts in visual navigation in insects, including aspects such as precise goal localization which are still not adequately known.

Another point of criticism concerns the analytical part of the study. Although I guess that many things can be explained without an overwhelming amount of tricky statistical tests, the results need to be quantified in some way and not remain a mere qualitative description of what happened. I am sure the author will be able to cope with these concerns in his revision of the text.

**Response:** We now have added panel H to Fig. 5, which shows the search distributions as notched boxplots which allow the reader to quickly assess significant differences between the distributions.

Comments to the authors

(1) You need to use your introduction to justify your experiments. Is there any evidence in the literature that the wasps might use other cues than visual surface structures for their homing behaviour. I could accept chemical cues (maybe brood scent), but auditory (from what?) and tactile cues (differences in sand structure?) seem a bit far fetched. But since you planned and executed the experiments, you might have some reasonable explanation. Please explain better why you think these cues are worth testing in your introduction.

**Response:** We now provide this explanation in the introduction (line 57ff): “Pinpointing the nest entrance, thus becomes a gradient descent in global image differences [43,44, see also 45-51] and experiments with large screens have added to other evidence (see examples in [1]) that navigating insects do make use of the full visual panorama and not only of individual landmarks [9]. However, whether it is the increasing salience of local visual cues that guide the final approach to the nest or whether pinpointing the nest location requires an ‘attentional’ switch to fine visual details, such as the nest entrance itself or visual features around it, is not known at present.

Moreover, in most cases it remains also unclear to what extent other cues help ground-nesting insects in their final approach to the nest. Desert ants, for instance, are guided by the CO^2^ plume emanating from the nest entrance [52] and other, very close-range cues such as local surface topography or even the sound of moving larvae emanating from the nest may help ground-nesting insects to pinpoint the exact location of the entrance. The role of such non-visual cues may be particularly important for insects such as the *Microbembex monodonta* we studied here, which return to a nest the entrance of which they had previously carefully closed, covered and camouflaged.”

(2) You say that the opaque barrier blocked distant visual cues. What were these distant cues? Please provide evidence that the wasps actually see these "distant cues" (depending on eye-morphology, some insects are very short-sighted).

Also interesting in this context is that they nest in "sandy areas with sparse vegetation" (line 47) and that your "study area was a mostly-bare, sandy blowout" (line 55). Thus distant cues might be extremely distant. Couldn't it be that the barrier just provided an additional landscape cue that the wasps did not "understand"?

**Response:** We now provide two additional figures in the supplementary material that show the wider visual environment at the study site (Fig. S1) and the visual background that was affected by the barrier in the experiment documented in Fig. 4. We also note that visual systems ’record’ all radiant (sky) and reflected light (terrestrial objects) irrespective of their distance. Insects are known to see the sun (ants, honeybees) and even the milkyway (dung beetles) which are both infinitely far away. We try to give an impression of what a wasp in our experiments might see of the scene around their nests by low-pass filtering views to approximately 1deg resolution (Fig. S1 & S2).

Alternative to your interpretation, the wasps might use solar compass (rough navigation to approach the site) and landscape structures for fine tuning of their homing and, eventually, surface cues for identifying the exact position. The new landscape cue you introduced might just have caused a general confusion in the wasps, and they abandoned the search. However, they should be used to changes in surface cues, since sand ripples are bound to change over time. Maybe you could discuss this topic.

**Response:** We now discuss the role of local and global visual cues in insect homing in the introduction and the discussion with reference to current research, in addition to the older literature (see response above), but also line 353ff in the discussion: “The accuracy with which panoramic views provide information on location depends on the distance of visual features [43,58] and on the sensitivity of insects to image differences. Close to the burrow, the image differences experienced by homing insects will depend on the visual salience of nearby objects, in terms of their angular size and contrast with the background.” and more extensively in line 385ff.

(3) You need to quantify your results. In your experiments on the exclusion of non-visual cues (lines 101-154) you show how this can be done. Why didn't you do the same (or something similar) in the other experiments? With these data you could even perform statistics (correct for pseudoreplication since you evaluated multiple events of the same wasp), which would be interesting particularly when looking at the digging attempt distribution of "olfactory cues only" against "visual surface cues only". There could actually be a statistical difference between these conditions.

**Response:** We show a bar graph for the barrier experiments in Fig. 4C and now have added a notched boxplot analysis for the search distributions in Fig. 5H for both the distribution of means and of the search spread.

(4**)** What was the purpose of Experiment 2 (lines 161 ff), which just repeats what you already tested before in Experiment 1 (I got a bit confused with the labelling of the conditions in the text, but in Figure 6 it is Fig. 6C and Fig.6F). Did you expect something "new" or different from this experiment? If so, why? If not, why did you perform the experiment?

**Response:** Justified in line 203ff: “The objective of this experiment was similar the previous, but offered an air-tight seal that robustly excluded both olfactory and auditory cues.”
